# Phenolic Profile, Antioxidant, Anti-Enzymatic and Cytotoxic Activity of the Fruits and Roots of *Eleutherococcus senticosus* (Rupr. et Maxim.) Maxim

**DOI:** 10.3390/molecules27175579

**Published:** 2022-08-30

**Authors:** Filip Graczyk, Jakub Gębalski, Anna Makuch-Kocka, Dorota Gawenda-Kempczyńska, Aneta A. Ptaszyńska, Sebastian Grzyb, Anna Bogucka-Kocka, Daniel Załuski

**Affiliations:** 1 Department of Pharmaceutical Botany and Pharmacognosy, Ludwik Rydygier Collegium Medicum, Nicolaus Copernicus University, Marie Curie-Skłodowska 9, 85-094 Bydgoszcz, Poland; 2 Department of Pharmacology, Faculty of Health Sciences, Medical University of Lublin, 4a Chodzki Str., 20-093 Lublin, Poland; 3 Department of Immunobiology, Institute of Biological Sciences, Faculty of Biology and Biotechnology, Maria Curie-Skłodowska University, Akademicka 19 Str., 20-033 Lublin, Poland; 4 College of Engineering and Health in Warsaw, Bitwy Warszawskiej 1920 r. 18 Str., 02-366 Warsaw, Poland; 5 Department of Biology and Genetics, Medical University of Lublin, 4a Chodzki Str., 20-093 Lublin, Poland

**Keywords:** *Eleutherococcus senticosus*, intractum, cytotoxicity, COVID-19, hyaluronidase, tyrosinase, polyphenols

## Abstract

*Eleutherococcus senticosus* (Rupr. et Maxim.) Maxim. is well-known for its adaptogenic properties in traditional Eastern medicine. It has been categorized as an endangered species due to the over-exploitation of the roots. As a result, alternatives must be found, including the usage of renewable aerial parts such as fruits. The goal of this research was to determine the phenolic compounds and the enzymatic, antioxidant, and cytotoxic activities of the intractum gained from the *E. senticosus* fruits and the mixture of chloroform-methanol roots extract with naringenin (3:7:5). The obtained results showed, that the intractum contained 1.02 mg/g ext. of polyphenols, 0.30 mg/g ext. of flavonoids, and 0.19 mg/g ext. of phenolic acids. In turn, the mixture of chloroform-methanol roots extract with naringenin (3:7:5) contained 159.27 mg/g ext. of polyphenols, 137.47 mg/g ext. of flavonoids, and 79.99 mg/g ext. of phenolic acids. Regarding the anti-enzymatic assay, the IC_50_ values for tyrosinase and hyaluronidase were equal to 586.83 and 217.44 [μg/mL] for the intractum, and 162.56 and 44.80 [μg/mL] for the mixture, respectively. Both preparations have possessed significant antioxidant activity in the ABTS, DPPH, and ferrozine tests. No cytotoxic effect on the FaDu and HEP G2 cancer cell lines was observed. Our findings support the traditional use of fruits and roots. Moreover, the results indicate also that adaptogens are rather nontoxic for normal and cancer cells, which corresponds with some hypotheses on adaptogens activity.

## 1. Introduction

The Araliaceae are a family of plants composed of approximately 1500 species of 43 genera. That family also contains the genus of *Eleutherococcus* Maxim. which includes about 40 species present in the areas of Northern Russia, China, Japan, and Korea. One of the most promising species is *Eleutherococcus senticosus* (Rupr. et Maxim.) Maxim. which in ancient times was commonly used in traditional medicine in northeastern Asia or eastern Russia. It is known for its adaptogenic activity [1,2].

Traditional Chinese medicine has known and used this plant for more than 4000 years, as a medicine to increase longevity. Europeans also knew the *E. senticosus*’s therapeutic properties, the oldest sources tell that in the mid-nineteenth century, the plant had become a subject of research by Russian scientists, with its peak in the 1970s, when it was used by USSR Olympians. It was reported, that in the 1970s Olympic Games, the Soviet contestants used a drink called Baikal, which was made of *E. senticosus* roots, to improve their sporting achievements. Later on, it gained more and more popularity over the years in central Europe [3,4,5,6].

Nowadays, it has become popular in Europe and the USA as a supplement for weaknesses, impotence, or immunity, mainly used as a dietary supplement and nutria-pharm product. *Eleutherococcus senticosus* turned out to be one of the ten most popular herbal supplements used in the United States, occurring in the forms of capsules, powdered plant material, or tea bags [7,8].

Over the years of its popularity, *E. senticosus* has been included in the group of so-called true adaptogens. In this group, *Eleutherococcus senticosus* has also been classified–*Eleutherococci radix et.rhizoma.* A majority of adaptogens are the roots usually of five–six year-old plants, which means that the plants are harvested as a whole or the roots are cut off and the plant needs a longer period for their regeneration. Therefore, it has been classified as a protected plant in some countries, such as South Korea. It is caused by overexploitation of the root material which has put the species in danger of extinction. For that reason, it is needed to find alternatives, in particular, the use of renewable aerial parts, e.g., the fruits, the aerial parts must be studied in detail to protect the species from overexploitation [9].

*Eleutherococcus senticosus* is rich in chemically different compounds, such as lignans, saponins, flavonoids, phenolic acids, and essential oil. Their main metabolites are termed the eleutherosides with eleutheroside B (syringin 4-β-D-glucoside) and eleutheroside E ((−)-siringaresinol 4,4′′-O-β-D-diglucoside), as main compounds. Eleutherosides have been found both in the underground and aerial parts of the plant. Furthermore, the fruits are rich in polyphenols (38.5–41.1 mg/g ext.), flavonoids (13.4–14.4 mg/g ext.), minerals (Mn 75.2–88.3, Fe 35.4–53.0, Zn 18.9–41.0, and Cu 3.34–13.0; mg/kg, respectively) [10,11,12].

The chemical diversity of plants is connected to their special biological properties. The *E. senticosus* extracts, mainly made from the roots, for many years have been used globally as an adaptogenic agent. Eleutheroside B and E were found to be responsible for the plant’s adaptogenic abilities [13]. It is proved that both eleutherosides present protective effects against nerve cell death, with a weak inhibitory effect on CYP2C9 and CYP2E1 but no effect on CYP2D6 and CYP3A4 [8]. Eleutheroside B was found to have cardioprotective abilities in rabbit heart models [14], while eleutheroside E is able to modulate the steroidogenesis, arachidonic acid, glutathione, and tyrosine metabolism in Winstar rats’ models. In turn, the fruits have been used in Russia as a food ingredient and a strengthening agent. However, it should be noted that in small doses, the powder fruits or infusion of it, have a reviving effect on the central nervous system, in higher dosages though, has a sedative effect [15].

Epidemiological studies carried out in the Soviet Union during the 1970s demonstrate that *E. senticosus* may reduce human mortality during influenza epidemics and typical complications of influenza infection, such as pneumonia, bronchitis, and otitis. Moreover, the morbidity rate has also been reduced, especially among children treated with a liquid extract for a month. Nowadays, the world is standing up to the coronavirus pandemic and many researchers are looking for a cure for tackling that. Coronavirus disease 2019 (COVID-19) is a pandemic pneumonia produced by the coronavirus that causes severe acute respiratory illness (SARS-CoV-2). The majority of severe COVID-19 patients experience difficulty breathing, and chest pressure, and eventually develop acute respiratory distress syndrome (ARDS), which has a high fatality rate. Infected lungs cause uncontrolled inflammation, which leads to fluid leakage and extracellular matrix accumulation. Hyaluronan (HA) is a component of the extracellular matrix (ECM) that has important physiologic and pathological functions. It is likewise found largely in the respiratory airways and increases in number during COVID-19 illness [16,17,18]. Nonetheless, there has been no obvious link between COVID-19 pathophysiology and HA yet.. A promising source of compounds with anti-COVID-19 activity might be plant-based compounds that act synergistically and are incomparable to those of synthetic compounds. Thus far, none of the plant compounds have progressed to the clinical stage related to anti-COVID-19. Based on the available data, long research stages are still required. 

Despite a large number of studies on the potential uses of plant-derived immunostimulators or adaptogens, few have progressed to the clinical stage. Considering *E. senticosus* some clinical studies were performed by Soviet scientists in the 1970s and 1980s. On the basis of our previous discoveries on the intractum from the *E. senticosus* fruits, we have concluded its potential adaptogenic activity [19], with possible immunostimulant and anti-inflammatory effects. It is also known that the compounds contained in the fruits and roots of *E. senticosus* can regulate the activity of the enzymes, such as hyaluronidase (Hyal) [20], which makes them potentially useful as preparations in COVID-19 treatment with anti-inflammatory activity. 

Regardless of this, there is a need to provide more information on whether it might be demonstrating any activities at the biochemical and cellular levels. Therefore, the question arises, whether preparations made of the fruits or roots of *E. senticosus* can affect the course of the disease caused by SARS-CoV-2 virus infection and if its mechanism is related to the inhibition of enzyme activity. To justify that thesis, the plant materials were examined in a series of in vitro assays for phytochemicals and bioactivities related to the analysis of phenolic compounds, antioxidant, enzymatic, and cytotoxic activity of the intractum from *E. senticosus* fruits, and the mixture of the chloroform-ethanol root extract with naringenin (3:7:5). Naringenin is a flavonoid with anti-inflammatory and immunomodulatory activities. 

In our work we have followed a new concept regarding plant extract, i.e., using polar and non-polar compounds extracted from one plant material, which allowed for an exhaustive use of the roots in terms of number and amount of extractables. The use of polar and non-polar constituents as a mixture is also a novel approach to the development of plant-based preparations. Polar and non-polar constituents with naringenin might act synergistically and, we suppose that potentially could increase their activity. Moreover, the plant material was harvested in Poland, which means that the species might possibly be introduced to crops in Poland to reduce the risk of its extinction. The plants imported from Russia or China and products made from them are very often of poor quality and do not meet the European requirements, so that is why this plant should be studied so extensively.

## 2. Results and Discussion

### 2.1. Chemical Composition

For clarity, the samples in this work were marked as follows: the intractum from the fruits as intractum; a combination of extracts from the roots (chloroform-methanol-naringenin) in a proportion 3:7:5 as an extract. 

It is known that plants produce dozens of compounds, which are responsible for the plant’s pharmacological activity and are incomparable to those of synthetic compounds. However, amongst a wide spectrum of phytochemicals present in one species, in many cases, only a small group of them is responsible for the activity. One of the most widespread groups of plant-based compounds is polyphenols. In the first phase of the research, we performed studies to provide information about the content of phenolic and polyphenolic compounds in the intractum and extract (Table 1). It appeared that the extract contains a higher amount of TPC, TFC, and total phenolic acids when compared to the intractum. All these differences are statistically significant with *p* < 0.001. The concertation of phenolic acids in the extract was almost half of the total phenolic components’ concentration; whilst the concertation of the flavonoids was almost equal to the concertation of total phenolic compounds.

It has been reported that the concentration of polyphenols and flavonoids in the *E. senticosus* fruits intractum was higher than that obtained in this study (130 and 92 mg/g ext., respectively). In addition to this, the TPC in the 75% ethanolic extracts from the fresh fruits of *Eleutherococcus* species growing in Poland, obtained using ultrasound-assisted extraction was also higher (6.1–19.7 mg/g dry sample). Furthermore, the concentration of TPC and TFC in the fruits did not alter during one-year storage in the ambient temperature. Probably it results from a different region of the plants’ growth. These fruits were collected in Bydgoszcz (N: 53°07′36.55″ E: 18°01′51.64″) compared to those earlier studied and collected in Rogów (51° 49′ N i 53′ E); [21,22].

There are few data on the phenolic acid content of *Eleutherococcus* species growing in Russia or Asia, as well as in their predicted environment. A good example of this is provided by Jang et al. [21] who found that the Korean *E. senticosus* fruit ethanol extract contained higher concentrations of polyphenols and flavonoids than that now estimated, i.e., 334.36 ± 12.8 mg/g ext. of TPC and 198.25 ± 23.5 mg/g ext. of TFC, respectively. Shoheal et al. [22], studied the EtOH and aqueous extracts of the *E. senticosus*; the study revealed the following concentration of flavonoids and polyphenols, 0.20%; 0.30%, and 0.30; 0.60%, respectively. 

Regarding the roots, the high content of phenolic compounds obtained in this study might result from the use of polar and non-polar solvents for extraction, which means that aglycons can be present in the extract. Adamczyk et al. [23] investigated the contents of flavonoids and polyphenols in the methanol root extract of *E. senticosus* and four other *Eleutherococcus* species. The TPC ranged from 4.10 ± 1.40 to 10.40 ± 1.30 and was expressed as gallic acid equivalents (mg GAE/g); TFC varied from 1.80 ± 0.02 to 6.50 ± 1.10 and was expressed as mg of quercetin equivalent (mg QEs/g). The obtained results in our study present higher concentrations of TFC and TPC in the extract made of *E. senticosus* roots, which may result from a combination of polar and non-polar extracts. 

There are plenty of results on TPC and TFC in plants, therefore we decided to contrast the obtained data only with other adaptogenic plants. A very popular species is *Schisandra chinensis* (Turcz.) Baill., the chemical composition of which was investigated by Mocan et al. [24]. Their results showed that *Schisandra chinensis* leaves and fruits were found to be a rich source of flavonoids, the ethanol leaves and fruits extracts presented sums of TPC–62.36 ± 1.38 mg GAE/g plant material, the fruits–9.20 ± 0.43 mg GAE/g plant material. Significantly, the TFC in both leaves and fruit extracts were lower–35.1 ± 1.23 mg rutin equivalents/g plant material and 7.65 ± 0.95 mg RE/g plant material resp. Jeong et al. [25] estimated that water fruit extract of the previously mentioned plant presented a TPC of 17.72 ± 0.39 mg GAE/g, and TFC of 33.43 ± 0.24 mg QE/g ext. Cheng et al. [26] researched the *Schisandra chinensis* pollen extract showing that it had high TPC (53.74 ± 1.21 mg GAE/g) and TFC contents (38.29 ± 0.91 mg rutin/g). 

Another adaptogenic plant–*Scutellaria baicalensis* Georgi was also used as an interesting point to counter-argue our results. Vergun et al. [27] estimated the total polyphenol, flavonoid, and phenolic acid content of *S. baicalensis* ethanol extracts, showing that leaves extract contained the highest amounts of those compounds–96.76 ± 2.18 mg GAE/g ext., 72.66 ± 2.69 mg QE/g ext. and 23.44 ± 1.13 mg CAE/g ext., resp. In turn, Li et al. [28] determined the phenolic content of the methanol *S. baicalensis* root extract, with the result of 36.30 ± 0.67 mg GAE/g ext. Chan et al. [29] reported that root extracts of *S. baicalensis* exhibited following TPC–water extract 3.85 ± 0.07 mg GAE/g; ethanol extract 3.65 ± 0.07 mg GAE/g; ether extract 3.45 ± 0.06 mg GAE/g). 

As can be noticed, the concentrations of polyphenolic compounds vary from themselves and surely are dependent on the type of plant material (underground or aerial part), the solvent used for extraction, extraction methods, and growth conditions. For this reason, it is important to control each batch of the harvested plant material to ensure the quality of the extract or plant-based preparations. 

### 2.2. Antioxidant Activity 

Antioxidants from plant sources are included in the human diet to protect the body at the molecular level. Three methods were included in the experiments. Testing different samples using different methods should show whether the antioxidative activity of plant samples depends on their chemical profile and the interactions with the free radical. The extracts’ antioxidant capacities were measured and expressed as the EC_50_ value. The different parts of the plant showed different antioxidant potency (Table 2). 

The ferrozine-based iron assay was used to study the metal chelating activity in the fruit and root preparations. The obtained results show that the extract is more abundant in iron chelating than the intractum, as well as is more active in ABTS and DPPH tests, with a statistical significance of *p* < 0.01. 

There is little data about the metal chelating of *E. senticosus*. Załuski et al. [30] report the chelating properties of 75% ethanol extract from the spring leaves, fruits, and roots from *E. senticosus*, proving metal chelating activity of the ethanol extracts from the roots and spring leaves (0.50 ± 0.05 mg/mL). The authors confirmed their contribution in another study, in which three *Eleutherococcus* species were examined, the outcomes revealed that extracts can bind Fe^2+^ with EC_50_ from 0.20 to 0.60 mg/mL, with 0.3 mg/mL for *E. senticosus* [31]. It is in line with our records, positively corresponding with chelating results of both the extract and the intractum. 

The literature provides some information about the antioxidative activity of other *Eleutherococcus* species. The methanol extracts of 5 *Eleutherococcus* species (*E. henryi, E. sessiliflorus, E. senticosus, E. gracilistylus, E. divariactus*) investigated by Adamczyk et al. [23] have shown influential antioxidant activities. Examined extracts at a concentration of 0.80 mg/mL, expressed DPPH reduction in the range of 14.7 to 26.2%, with *E. gracilistylus* as the most active species. In turn, Liu et al. [32] have investigated the potential of chemical compounds possessed by *E. senticosus*, such as flavonoids, and phenolic acids, and correlated it with its potential to reduce DPPH and ABTS, proving that high concentrations of those components are positively correlated with high antioxidant activity. Those findings stand in line with our current and previous reports [19].

*Schisandra chinensis* (Turcz.) Baill. was analyzed to compare our results with this adaptogenic plant. Mocan et al. [24] have also examined the antioxidant activities of this plant. For the *S. chinensis* leaves extracts, the DPPH assay was 26.87 ± 0.84 µg QE/mg plant material, and the ABTS assay–45.97 ± 0.31 µg Trolox equivalents (TE)/mg plant material. In the case of *S. chinensis* fruit extracts, the results were 7.80 ± 0.55 µg QE/mg plant material and 15.95 ± 0.68 µg TE/mg plant material, respectively. The results obtained by Jeong et al. [25] showed that water extracts of *S. chinensis* fruits did also exhibit inhibition of DPPH, 26.37 ± 2.42%, however, it did not show any ion-chelating activities. In turn, Cheng et al. [26] reported the capacity of *S. chinensis* pollen extracts to chelate iron which was equal to 23.24 ± 0.79 mg Na_2_EDTA per gram extract.

To counter-argue with another plant, Vergun et al. [27] estimated an antioxidant ability of *S. baicalensis* G. leaves, with results in the range of 7.63–8.83 mg Trolox equivalents (TE)/g ext. Li et al. [28] determined the antioxidant capacity of whole plant methanol extracts, with results of 184.34 ± 4.50 μmol Trolox/g ext. Li et al. provided a set of results regarding an antioxidant activity of *S. baicalensis* extracts with EC_50_ at 70.81 and 35.34 μg/mL in DPPH and ABTS respectively. Chanaj-Kaczmarek et al. [33] investigated the ion-chelating properties of *S. baicalensis* hydroalcoholic root extracts, with the EC_50_ results of 8.54 ± 0.013 mg/mL. The obtained data suggest that the high antioxidant activity of researched plants might be paired with their adaptogenic abilities. 

### 2.3. Enzymatic Activity

All the results obtained are presented as a IC_50_ value (Table 3). Both the intractum and extract showed inhibitory activity against tyrosinase (TYR) and hyaluronidase (Hyal). However, the extract presented, statistically significantly (*p* < 0.001), much lower concentrations needed to inhibit both tyrosinase and hyaluronidase compared to the intractum. 

Inflammation is a natural process that is induced by a variety of stimuli, including hyaluronidase or tyrosinase. Hyaluronidases are enzymes that break down hyaluronic acid. Many researchers have established a link between the expression of Hyal and the increase of inflammation and tumor growth. It was discovered that its activity is increasing in a variety of cancer diseases. Therefore, the identification and classification of Hyal inhibitors might be beneficial in the development of anti-inflammatory drugs. The intractum or extract have already been shown to have anti-enzymatic action. 

Intractum inhibited hyaluronidase by 50% at a concentration of 217.44 ± 10.72 [μg/mL], while tyrosinase was inhibited at the same level at a concentration of 586.83 ± 2.36 [μg/mL]. Simultaneously, the extract inhibited hyaluronidase and tyrosinase by 50% at a concentration of 44.80 ± 3.11 and 162.56 ± 0.02 [μg/mL], respectively. Compared to the reference substances, the research shows that both samples are greater at inhibiting Hyal than the standard substance aescin, the extract showed the strongest inhibition. These differences are statistically significant with *p* < 0.001. This means it could have potential use as a healing substance. 

Unfortunately, the research literature on the anti-enzymatic activity of *Eleutherococcus* is rare, and it is hard to make a comparison. There are just a few reports on that provided by Adamczyk or Kuźniewski. Adamczyk et al. [23] investigated the antiacetylcholinesterase and antihyaluronidase activities of *E. senticosus* root extracts, at the concentration of 100 μg/0.16 mL, obtaining the results of 26.10 ± 0.5% inhibition and 10.40 ± 0.60% inhibition, respectively. Kuźniewski et al. [34] found that 75% ethanol extracts from the fall and early spring leaves of *E. senticosus* inhibited Hyal at 74.3 and 33%, respectively, at a concentration of 22 μg/0.16 mL of the reaction mixture. The increased inhibition of autumn leaves extracts may be due to the presence of more polyphenolic substances like tannins. 

Liyanaarachchi et al. [35] studied the potential of the 15 ethanol extracts to inhibit hyaluronidase and tyrosinase. Only eight extracts were found to show such an action in the range of 34.8 to 95 percent inhibition. *Curcuma aromatica* rhizoma was the most effective–95.0% inhibition of hyaluronidase at 500 μg/mL; *Artocarpus altilis* and *Artocarpus nobilis* bark extracts inhibited tyrosinase (IC_50_ 27.47 ± 0.45 μg/mL, 53.23 ± 2.65 μg/mL, respectively) and hyaluronidase (68.59%, 44.78% inhibition at 500 μg/mL, respectively). Neimkhum et al. [36] report that the ethanolic *Carissa carandas* extracts from different parts showed a high hyaluronidase inhibition, leaves 97.8 ± 1.6%, and fruit 83.9 ± 2.2%, respectively. In turn, the ethyl acetate extracts from *C. carandas* Linn. have great anti-tyrosinase potential, fruit and leaves extracts inhibited the enzyme in 47.3 ± 4.2% and 47.7 ± 7.1%, resp. 

The anti-hyaluronidase activity of aqueous extracts from 12 plant species was studied by Piwowarski [37]. Amongst them, *Lythrum salicaria* L. inhibited the strongest Hyal (64.9%), while *Geum urbanum* L., *Rubus ideus* L., and *Quercus robur* L. had lesser inhibition (25.6%; 21.2%; 20.2%, respectively). According to [38], the methanol extract from the bark of *Schotia brachypetala* inhibits Hyal at 75.1%, while the methanol extract from the leaves of *Psychotria capensis* inhibits Hyal at 52.9%. It is noteworthy that some of the aqueous leaf extracts of common medicinal and culinary plants have been found to be effective, *Rosmarinus officinalis* L., *Origanum majorana* L., and *Ocimum basilicum* L. have inhibited hyaluronidase at the level of 100, 89 and 78%, respectively [39].

To compare our findings with another adaptogenic plant, we looked for data about *S. chinensis* (Turcz.) Baill. Mocan et al. [40] examined the anti-tyrosinase effect of leaves and fruits methanol extracts (15.53 and 10.24 mg kojic acid equivalents (KAE)/g extract). In turn, Choi et al. [41] discovered that ethyl acetate extracts of *S. chinensis* fruits were able to inhibit hyaluronidase (100 U/mL) with the IC_50_ of 2.50 mg/mL. Cha [42] conducted a study, where high anti-enzymatic abilities of methanol *S. chinensis* extracts were confirmed, tyrosinase (220 U/mL) was inhibited by 75.1%, and hyaluronidase (7900 U/mL) was inhibited at 3.2%. 

*Scutellaria baicalensis* Georgi is another interesting adaptogenic plant. Chanaj-Kaczmarek et al. [33] investigated the hyaluronidase-inhibition properties of its hydroalcoholic radix extracts, with the IC_50_ value of 2.00 ± 0.06 mg/mL. Hong et al. [43] discovered that the *S. baicalensis* extracts contain compounds inhibiting tyrosinase (using B16F1-cell assays and tyrosinase activity assays). 

There are a lot of results regarding the anti-enzymatic activity of plant-based extracts or metabolites. However, there are also some limitations when it comes to the comparison of the results. It results from the different enzyme activity units used in different experiments and types of extracts. In many cases, the data on plant growth conditions and the method of drying are not provided. Another obstacle is a lack of an appropriate control, in the case of hyaluronidase aescin is recommended, while for tyrosinase kojic acid. We incorporated the enzyme activity unit in the text where it was provided.

### 2.4. Cytotoxicity

In this study, we have postulated that the intractum and extract may play a physiological role in inhibiting some cancer cell lines. However, the Russian scientific reports have revealed that adaptogens should not act as a cytotoxic agent toward cancers but should stimulate non-specific body resistance, as an adaptogenic stimulant. This theory was based on the knowledge considering the medicinal usage of the *E. senticosus* fruits by the Russians living in Siberia or for the Russian Olympians, as to increase their immunity and physical performance. When it comes to cancers, no direct action on cells should take place but only stimulation of the immune system to destroy cancer cells. To confirm that, we have chosen as an example, two cancer cell lines, FaDu and HepG2.

FaDu is a cell line with epithelial morphology that was recognized from a punch biopsy of a hypopharyngeal tumor patient with squamous cell carcinoma. This cell line is suitable for cancer and immuno-oncology research. HEPG2 (HepG2) cells are human liver cancer cell lines. Because of high cell specialization (both functional and morphological), the HepG2 and FaDu cells are a suitable model to study cytotoxic activities of xenobiotics in vitro and are appropriate to examine the toxicity [44,45,46].

**Figure 1:**
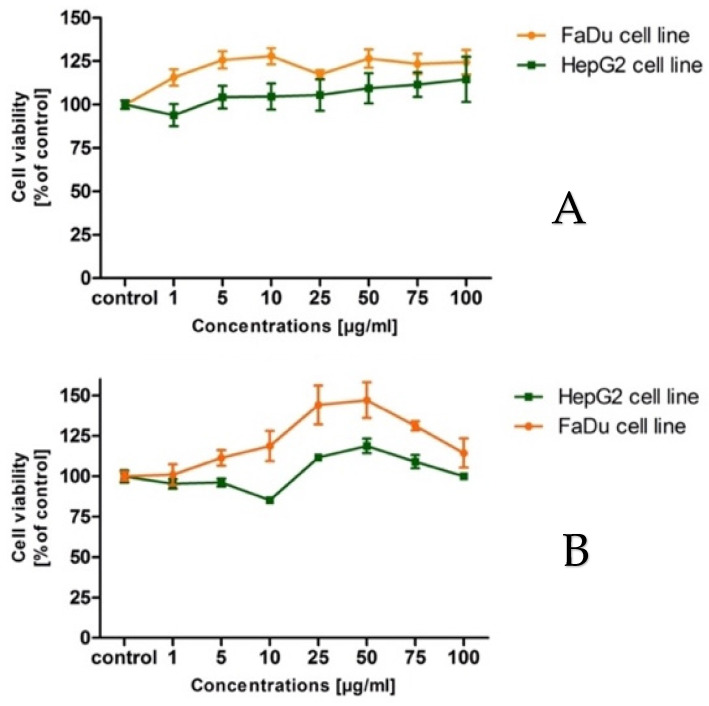
The cytotoxic activity of the samples towards FaDu and HepG2 cell lines. (**A**) cell viabilities with the intractum; (**B**) cell viabilities with the extract. Extract concentration 1, 5, 10, 25, 50, 75, 100 μg/mL.

As shown in Figure 1, both cell lines’ viability after being treated with the intractum and the extract, not only reveals the lack of lowering the cell lines’ viabilities, but also a slight increase (Figure 1A,B). The plant and its products should not cause any cytotoxic effect in the living cells in vitro or in vivo.

The cytotoxic assay has shown that both examined samples did not affect cancer cell lines to decrease its viabilities. It is in accordance with the knowledge, that adaptogenic plants and preparations cannot be harmful to living organisms, even cancer cells. It should not cause any toxic effects in the recommended doses, and that regards both healthy and cancer cells. In the case of cancer cells, in the 1960s and 1970s, it was postulated that *E. senticosus* should not directly inhibit cancer cells’ growth, but induce and stimulate the immune system to destroy cancer cells. This is in consent with the latest findings by Graczyk et al. [19], who found that *E. senticosus* fruit intractum is non-toxic toward A549 cancer cell lines at a concentration of up to 300 μg/mL. These findings stay in line with our current findings, pointing out that in the case of cancer diseases their administration should be careful and cautious.

Some authors reported cytotoxic activity of the adaptogens toward cell lines [47,48]. It has been reported that the silver nanoparticles of the *Eleutherococcus senticosus* dried stem parts demonstrated substantial cytotoxic and apoptotic effects on A549 lung cancer cells and HT29 colon cancer cells at a concentration of 10 μg/mL. These findings imply that the anticancer potential of Sg-AgNPs is linked to the stimulation of apoptosis via the Caspase-3/p38 MAPK pathway through the creation of ROS [49].

Załuski et al. [50] gave a report about the cytotoxic properties of the *E. senticosus* intractum on five leukemic cell lines, HL-60, HL-60/MX1, HL-60/ MX2, CEM/C1, and CCRF/CEM, informing that the IC_50_ value was in a range of 10.40–50 μg/mL. The author also contributed research, informing about the cytotoxic properties of different *E. senticosus* parts’ extracts the IC_50_ values for HL-60 were determined using ethanol extracts from roots, spring and fall leaves at concentrations of 208, 312, and 299 μg/mL [51]. That is further evidence that adaptogens are rather weak anticancer agents and do not directly inhibit the cells’ growth.

To compare, another study of an alternative adaptogenic plant–*Schisandra chinensis* proved that the lignans present in fruits present anti-cancer effects by inducing G0/G1 cell cycle arrest in A2780, ovarian cancer cells and decreasing the pro-tumoral phenotype of tumor-associated macrophages (TAMs). The study showed that the IC_50_ value of 27.81 μM for deoxyschizandrin, the main lignan present in *Schisandra* berries, inhibited cell growth [52]. Another result, delivered by Chung et al. [53] tells that ethanol berry extracts of *S. chinensis* in the concertation up to 400 μg/mL, did not exhibit any effect on cell viability of the HepG2 cancer cell lines. That news was also confirmed by Sung et al. [54].

*Scutellaria baicalensis* is another adaptogenic herb that was also brought up as a counter-argument to our disclosures. Cho et al. [55] estimated with the MTT assay that methanolic extracts exhibited significantly low cytotoxicity at 100 μg/mL and 10 μg/mL on B16 melanoma cells. Ye et al. [56] provided the data that *S. baicalensis* is able to inhibit hepatocellular carcinoma cell growth in vitro. However, that plant extract exhibited a weak decrease in the cell viability of the HepG2 cells with IC_50_ of 360 μg/mL. Previous reports of this author can also confirm the weak *Scutellaria baicalensis* anticancer abilities–plant water extract proved an inhibition of growth in various cancer cell lines (HepG2, MCF-7, PC-3, LNCaP, KM-12, HCT-15, KB and SCC-25) with the IC_50_ of 1.1, 0.90, 0.52, 0.82, 1.1, 1.5, 1.0, 1.2 mg/mL, respectively [57].

Very high concentrations were needed, regardless of whether normal or cancer cells were used, so it is in agreement with the role of an adaptogen, which is thought to be a rather nontoxic substance for normal cell lines and according to some hypotheses, for cancer cells as well [58,59]. Moreover, it is significant that adaptogenic drugs should increase the general immunity of the living organism. Our previous reports show that the intractum promoted peripheral blood leukocytes (PBLs) to increase their proliferation. The increase in the number of leukocytes is in a line with our hypothesis on the adaptogenic activity of the fruits and their preparations [19].

## 3. Materials and Methods

### 3.1. Chemicals and Reagents

The standards of quercetin ≥ 95.0%, aescin ≥ 95.0%, donepezil ≥ 98.0%, kojic acid ≥ 97.0%, ascorbic acid ≥ 99.0%, gallic acid ≥ 97.5%, caffeic acid ≥ 98.0%, were obtained from Merck (Darmstadt, Germany). The sodium carbonate ≥ 99.0%, potassium acetate ≥ 99.0%, were obtained from Sigma-Aldrich (St. Louis, MO, USA). The sodium molybdate, sodium nitrate, ethyl acetate, hydrochloric acid, sodium hydroxide, aluminum chloride, ferrous chloride, potassium peroxodisulfate were purchased from POCH (Gliwice, Poland).

Methanol (MeOH), ethanol (EtOH) was purchased from J.T. Baker (Phillipsburg, NJ, USA). Ultrapure water was prepared using a Millipore Direct-Q3 purification system (Bedford, MA, USA).

1,1-diphenyl-2-picryl-hydrazyl (DPPH), DMSO, 2,2′-azino-di-(3-ethylbenzthiazoline sulfonic acid) (ABTS), 3-(2-Pyridyl)-5,6-diphenyl-1,2,4-triazine-p,p′-disulfonic acid monosodium salt hydrate (FerroZine Iron Reagent), bovine albumin, hyaluronidase (Hyal) from bovine testes type I-S, hyaluronic acid, cetyltrimethylammonium bromide (CTAB), 5,5′-dithiobis(2-nitrobenzoic acid (DTNB), acetylcholinesterase (AChE), acetylthiocholine iodide (ACTI), tyrosinase, L-tyrosine, acetate buffer pH 5.35, sodium phosphate buffer pH 7.0, phosphoric buffer pH 6.8, phosphoric buffer pH 7.5 were obtained from Sigma-Aldrich.

FaDu and HepG2 cell lines were obtained from American Type Culture Collection (Manassas, VA, USA), cultured with EMEM (Sigma Aldrich, Saint Louis, MO, USA) supplemented with 10% fetal bovine serum (Sigma Aldrich, Saint Louis, MO, USA), 100 U/mL of penicillin and 100 mg/mL of streptomycin (PenStrep, Sigma Aldrich, Saint Louis, MO, USA). All other reagents were of analytical grade or higher. 

### 3.2. Plant Material and Preparation of the Extracts

The matured fruits and roots of *Eleutherococcus senticosus* (Rupr. et Maxim.) Maxim. were collected at the Garden of Medicinal and Cosmetic Plants in Bydgoszcz (Poland) in September 2020 (N: 53°07′36.55″ E: 18°01′51.64″). The plant sample was deposited at the Department of Pharmaceutical Botany and Pharmacognosy, Collegium Medicum, Bydgoszcz, Poland. The roots after drying were stored under domestic conditions (room temperature, paper bag, dark place). 

I: The intractum was prepared according to the method previously described by Graczyk el. al (20). Plant materials’ identity was evaluated morphologically and by HPLC-DAD and HPLC-RID analysis, in comparison with reference data. The fresh fruits (20 g) were macerated in 100 mL 40% ethanol for 30 days under domestic conditions (ambient temperature, sunless place). After that, the extract was filtered through Whatman no. 4 filter paper. The solvent was dried with an evaporator under vacuum conditions at 45 °C, next frozen at −20 °C and subjected to lyophilization. The dried residue was stored in an exicator at 4 °C. Sample, for the further analysis, was marked as intractum.

II: The mixture of the chloroform-ethanol extract with naringenin (3:7:5); (combination of lipophilic and hydrophilic active compounds and naringenin). The air-dried roots (5 g each) were soaked in 50 mL 75% methanol for 24 h. Next, the samples were subjected to triple UAE type extraction (ultrasonic bath, Polsonic, Warsaw, Poland) using 1 × 50 mL and 2 × 25 mL of 75% ethanol. The extraction was performed at room temperature for 15 min for each cycle. Finally, 100 mL of each extract was obtained. The solvents were dried with an evaporator under vacuum conditions at 45 °C, next frozen at −20 °C and subjected to lyophilization. The dried residue was stored in an exicator at 4 °C. To obtain the chloroform extract, the roots were extracted in the same way as an extraction with 75% methanol using chloroform. After that, the extracts were combined in a proportion 3:7:5 (chloroform-methanol-naringenin). Sample, for the further analysis, was marked as extract. 

### 3.3. Chemical Composition Assays

#### 3.3.1. Folin-Ciocalteu Method for Polyphenols (TPC)

The Folin–Ciocalteu (F-C) reaction is an antioxidant assay, which measures the reductive capacity of an antioxidant, and is a spectrophotometric method based on the ability of polyphenols to form colored reactions with the Folin-Ciocalteu reagent [60]. The absorbance is proportional to the total content of phenolic compounds in the tested sample [61]. To achieve optimal conditions for our study, the following parameters were used, 25 μL of the extract was mixed with 25 μL of Folin-Ciocalteu reagent. The solution was kept at 25 °C for 5–8 min before adjusting the volume to 200 μL with distilled water and adding 25 μL of sodium carbonate solution. After 60 min, the absorbance was measured at a wavelength of 750 nm with a spectrophotometer. Gallic acid was used as standard for the calibration curve. Total phenolic content was determined with the calibration curve for gallic acid as mg gallic acid equivalents per gram of extract (mg/g).

#### 3.3.2. Total Flavonoids Content (TFC)

The amounts of total flavonoids were determined by Christ and Müller’s aluminum chloride method and measured by a colorimetric assay [62]. The original method was adapted to our research with the following factors: 25 μL of examined samples were added to 75 μL of ethanol. Then, 10 μL of 1M potassium acetate was added. After 5 min, 10 μL of 10% aluminum chloride was added. Immediately, the mixture was diluted by the addition of 130 μL distilled water and mixed thoroughly. The absorbance was determined at 510 nm versus a blank after incubating for 30 min in the darkness. Quercetin was used as standard for the calibration curve. Total flavonoids content of the extract was expressed as mg quercetin equivalents per gram of extract (mg/g).

#### 3.3.3. Total Phenolic Acids Content (TPAC)

Determination of total phenolic acid content by the Arnov method, which is a spectrophotometric method utilizing Arnov’s reagent, described by Gwalik-Dziki [63]. The method was adapted to our study with undermentioned parameters: 25 μL of the extracts was mixed with 150 μL of water, 25 μL of hydrochloric acid (18 g/L), 25 μL of Arnov’s reagent (10 g of sodium molybdate and 10 g of sodium nitrate dissolved in 100 mL of methanol) and 25 μL of sodium hydroxide solution (40 g/L). Phenolic acid content was calculated as caffeic acid percent measured at wavelength of 490 nm. 

### 3.4. Antioxidant Activity Assays

#### 3.4.1. ABTS Assay

Numerous methods are used to evaluate antioxidant activities of natural compounds in plant-based drugs with varying results. One of them used for determining the antioxidant activity of extracts is using bleu/green ABTS reagent. ABTS assay measures the relative ability of antioxidants to degenerate the ABTS in aqueous phase, as compared with standard [64,65]. Based on the original method, 10 μL of examined samples were incubated for 30 min with 190 μL of ABTS solution in the darkness. Absorbance was measured at wavelength of 734 nm. As the reference compound, ascorbic acid was used. The results were estimated using the following equation:

(1) $${\%}_{\mathrm{ABTS}}=(\frac{A_{\mathrm{blank}}-A_{\mathrm{sample}}}{A_{\mathrm{blank}}})\;\times \;100\%$$

A_blank_—absorbance on the blank

A_sample_—absorbance of the sample

#### 3.4.2. DPPH Assay

DPPH (2,2-diphenyl-1-picryl-hydrazyl-hydrate) is another method used to measure the antioxidant capacity of examined samples. Its mechanism is based on losing the absorption of violet DPPH solution to colorless in the presence of antioxidant molecules in examined samples [64,66]. To optimize the original method for our research, the following parameters were used: 10 µL of extracts combined with 280 µL of DPPH solution, added 10 µL of methanol, the absorbance was measured for 1 h at 515 nm. As the reference compound, ascorbic acid was used. The results were calculated using the following equation:

(2) $$\mathrm{DPPH}=(\frac{(A_{\mathrm{control}}-\left(A_{\mathrm{sample}}-A_{\mathrm{blank}}\;\right)}{A_{\mathrm{control}}})\;\times \;100\%$$

A_control_—absorbance DPPH

A_sample_—absorbance extract/standard

A_blank_—absorbance MeOH

#### 3.4.3. Ferrozine Assay

The iron colorimetric assay was performed to determine the chelating abilities of the iron in diagnosed samples [67,68]. To optimize the method for our research, 75 μL of examined plant extracts samples were incubated for 5 min at 25 °C with 15 μL of FeCl_2_ solution, topped up to volume 200 μL of reaction mix with ethanol. After that, 15 μL of ferrozine solution was added and incubated for 5 min and temperature 25 °C. 

As a result, the reduced ferrozine complex causes blue color. Sample iron percentage of chelation is determined by comparing the 560 nm absorbance of sample wells to the absorbance of ascorbic acid. The results were estimated using the following calculation:

(3) $${\%}_{\mathrm{CHELATING}\;\mathrm{EFFECT}}=(\frac{A_{\mathrm{blank}}-A_{\mathrm{sample}}}{A_{\mathrm{blank}}})\;\times \;100\%$$

A_blank_—absorbance on the blank

A_sample_—absorbance of the sample

### 3.5. Enzymatic Activity Assays

#### 3.5.1. Tyrosinase Assay

In 96-well plates, tyrosinase inhibitor experiments were carried out using a modified method given by Sigma-Aldrich (Saint Louis, MO, USA) [69]. Tyrosinase is the enzyme that converts L-tyrosinase to L-DOPA and L-DOPA to DOPA-quinone, causing the solution to become brown. 10 μL of sample (1 mg/mL), 140 μL of phosphoric buffer (pH = 6.8), and 25 μL of an enzyme (125 U/mL in phosphoric buffer pH = 6.8) were combined and incubated at room temperature for 10 min. A control lacking the inhibitor was also prepared (Ac). Following incubation, 25 μL of L-tyrosine (0.3 mg/mL) was added to each well, and the absorbance was measured at 510 nm (kinetic model, every 5 min). The graph’s linear range was then divided into two time points (t1 and t2). All samples were tested three times. The kojic acid (0.01 mg/mL) was used as a standard. The tyrosinase inhibition was calculated using the following equation:

(4) $${\%}_{\mathrm{INHIBITION}}\;=\frac{{\Delta A}_{c}-{\Delta A}_{s}}{{\Delta A}_{C}}\;\times \;100\%$$

A_C_—the difference in absorbance between time T2 and T1 for positive control

A_S_—the difference in absorbance between time T2 and T1 for sample

#### 3.5.2. Hyaluronidase Assay

The hyaluronidase inhibitor screening assay (Sigma Aldrich, Saint Louis, MO, USA) is a two-step turbidimetric reaction, measuring the amount of hyaluronic acid that is hydrolyzed by the enzyme. The decrease in turbidity is proportional to the enzymatic activity in the sample [70].

The assay was performed, accordingly to the method, in 96-well plates, by precipitating non-hydrolyzed hyaluronic acid (HA) with cetyltrimethylammonium bromide (CTAB), the activity of the inhibitors was measured. 10 μL of sample (0.5 mg/mL), 15 μL of acetate buffer (Ph = 5.35), 25 μL of incubation buffer (pH = 5.35, 0.1 mg/mL BSA, 4.5 mg/mL NaCl), and 25 μL of enzyme (30 U/mL, incubation buffer) were mixed together. After a 10-min incubation at 37 °C, a hyaluronic acid solution of 25 μL (0.3 mg/mL in acetate buffer pH = 5.35) was added. Plates were then incubated at 37 °C for 45 min. Non-hydrolyzed HA was precipitated by adding 200 μL of 2.5% CTAB after incubation. For 10 min, the plates were maintained at 25 °C. At 600 nm, the intensity of complex formation was measured. The absorbance of solution without inhibitor (AC) and enzyme (AT) were tested to evaluate the existence of inhibition. All samples were tested three times. As a reference, aescin (0.01 mg/mL) was used. The inhibition of hyaluronidase was determined using the following equation:

(5) $${\%}_{\mathrm{INHIBITION}}\;=\frac{A_{S}-A_{C}}{A_{T}-A_{C}}\;\times \;100\%$$

A_S_—absorbance of the HA + sample + enzyme

A_C_—absorbance of the HA + enzyme

A_T_—absorbance of the HA + sample 

#### 3.5.3. Acetylcholinesterase Assay

Acetylcholinesterase (AChE) catalyzes the hydrolysis of the acetylcholine into choline and acetic acid, the inhibitor screening kit (Sigma Aldrich, Saint Louis, MO, USA) method, which is produced by the action of AChE choline presents a yellow color with 5,5′-dithiobis (2-nitrobenzoic acid) (DTNB), was used. The intensity of the product color is proportional to the enzyme activity in the investigated samples [71]. The assay was performed with 96-well plate, with spectrophotometric multiwell plate reader. 45 μL of enzyme (0.4 U/mL, pH = 7.5 phosphoric buffer) and 5 μL of sample (0.1 mg/mL) were combined and incubated at room temperature for 15 min. After incubation, 150 μL of solution (154 μL of buffer, 1 μL of substrate, and 0.5 μL of DNTB) were added, and absorbance was measured at two points, t0 and t10, at 405 nm. All samples were tested three times. As a standard, physostigmine (0.05 mg/mL) was used. The following equation was used to compute tyrosinase inhibition:

(6) $${\%}_{\mathrm{INHIBITION}}\;=\frac{1-A_{s}}{A_{C}}\;\times \;100\%$$

A_S_—absorbance of the acetylcholine + enzyme + sample

A_C_—absorbance of the acetylcholine + enzyme 

### 3.6. Cytotoxicity Screening Assay 

FaDu and HepG2 cell lines were obtained from American Type Culture Collection (Manassas, VA, USA) and cultured using EMEM (Sigma Aldrich, Saint Louis, MO, USA) supplemented with 10% fetal bovine serum (Sigma Aldrich, Saint Louis, MO, USA), 100 U/mL of penicillin and 100 mg/mL of streptomycin (PenStrep, Sigma Aldrich, Saint Louis, MO, USA). Cell lines were incubated at 37 °C in a humidified atmosphere of 5% CO_2_. Cytotoxic effects of tested extracts and reference substances were examined using MTT assay (Sigma Aldrich, Saint Louis, MO, USA) [72] according to the manufacturer’s procedure. Stock solutions of the tested compounds were prepared dissolving in a sterile DMSO. The suspension of cells was prepared at a density of 1 × 10^5^ cells/mL. Cells were incubated for the next 24 h with different concentrations of the tested compounds (1–100 μg/mL). The absorbance was measured at 570 nm using a microplate reader (Epoch, BioTek Instruments, Santa Clara, CA, USA). Each experiment was repeated three times. Results are shown as % of controls cells.

### 3.7. Statistical Analysis

The results were expressed as the mean ± SEM of three independent experiments performed at least in triplicate. The obtained data were subjected to statistical analysis using Statistica 13.1 (StatSoft, Cracow, Poland). Statistical differences between content of phenolic and polyphenolic compounds in the intractum and extract were estimated by Student’s *t*-test. ANOVA test was performed to compare the antioxidant and anti-enzymatic activity of the intractum, extract and reference substance. The Scheffe test was used as a post-hoc analysis. All statistical tests were carried out at significance level of *p* = 0.05. 

## 4. Conclusions

To summarize, our research shows for the first time that the *E. senticosus* fruits and polar and non-polar roots extract, which have traditionally been used as medicine in Russia and China, are a source of secondary plant metabolites, with an anti-hyaluronidase and anti-tyrosinase activity.

The results obtained indicate also that adaptogens are rather nontoxic for normal and cancer cells, which corresponds with some Soviet hypotheses on adaptogens activity. However, those have yet to be explored by means of modern metabolomics techniques, such as HPLC-MS or NMR. Simultaneously, it must be noticed that plant-based extracts in some cases should not be administered without health testing. More studies in in vitro and in vivo models evaluating a deep phytochemical analysis, bioavailability, toxicity, and also metabolomics-oriented investigations are required and will be included in our future studies, therefore it could help further our understanding of those molecules.

## Figures and Tables

**Table 1:** Chemical composition of the *E. senticosus* intractum and the extract [mg/g ext. ± SD]. Different superscript letters within the same row indicate statistically significant differences with *p* < 0.001.

	*Intractum*	Extract
Total Phenolic Compounds	1.02 ± 0.04 ^a^	159.27 ± 2.73 ^b^
Total Phenolic Acids	0.19 ± 0.05 ^a^	79.99 ± 3.57 ^b^
Total Flavonoids Content	0.30 ± 0.07 ^a^	137.47 ± 5.23 ^b^

**Table 2:** Antioxidant activity of the intractum and extract, EC_50_ value presented as [μg/mL]. * % Chelating of iron was presented in %. Ascorbic acid was used as a reference substance. Different superscript letters within the same row indicate statistically significant differences with *p* < 0.01.

Method	*Intractum*	Extract	Ascorbic Acid
Ferrozine Assay *	12.12 ± 1.86 ^a^	26.34 ± 1.14 ^b^	6.44 ± 0.02 ^c^
ABTS	55.22 ± 1.15 ^a^	18.10 ± 0.20 ^b^	2.27 ± 0.07 ^c^
DPPH	250.48 ± 19.99 ^a^	138.17 ± 4.28 ^b^	24.93 ± 0.28 ^c^

**Table 3:** The anti-enzymatic activity of the intractum and extract. The results are presented as the IC_50_ value [μg/mL]. * Kojic acid was used as a reference substance in tyrosinase assay, physostigmine in acetylcholinesterase assay and aescin in hyaluronidase assay. Different superscript letters within the same row indicate statistically significant differences with *p* < 0.001.

Enzyme Unit Activity (U/mL)	*Intractum*	Extract	Reference Substance *
**Tyrosinase** (125)	586.83 ± 2.36 ^a^	162.56 ± 0.02 ^b^	44.43 ± 0.23 ^c^
**Acetylcholinesterase** (0.4)	N/A	N/A	5.00 ± 0.11
**Hyaluronidase** (30)	217.44 ± 10.72 ^a^	44.80 ± 3.11 ^b^	388.80 ± 3.45 ^c^

## Data Availability

Not applicable.
